# Crystal structure of a complex of human chymase with its benzimidazole derived inhibitor

**DOI:** 10.1107/S0909049513020748

**Published:** 2013-09-25

**Authors:** Yoshiyuki Matsumoto, Shinji Kakuda, Masahiro Koizumi, Tsuyoshi Mizuno, Yumiko Muroga, Takashi Kawamura, Midori Takimoto-Kamimura

**Affiliations:** a Teijin Institute for Bio-medical Research, 4-3-2 Asahigaoka, Hino, Tokyo 191-8512, Japan

**Keywords:** chymase inhibitor, benzimidazole, crystal structure, FMO calculation

## Abstract

The crystal structure of human chymase complexed with a novel benzimidazole inhibitor, TJK002, was determined at 2.8 Å resolution. The X-ray crystallographic study shows that the benzimidazole inhibitor forms a non-covalent interaction with the catalytic domain of human chymase. The hydrophobic fragment of the inhibitor occupies the S1 pocket. The carboxylic acid group of the inhibitor forms hydrogen bonds with the imidazole N(∊) atom of His57 and/or the O(γ) atom of Ser195 which are members of the catalytic triad. This imidazole ring of His57 induces π–π stacking to the benzene ring of the benzimidazole scaffold as P2 moiety. Fragment molecular orbital calculation of the atomic coordinates by X-ray crystallography shows that this imidazole ring of His57 could be protonated with the carboxyl group of Asp102 or hydroxyl group of Ser195 and the stacking interaction is stabilized. A new drug design strategy is proposed where the stacking to the protonated imidazole of the drug target protein with the benzimidazole scaffold inhibitor causes unpredicted potent inhibitory activity for some enzymes.

## Abstract 2

The crystal structure of human chymase complexed with a novel benzimidazole inhibitor, TJK002, was determined at 2.8 Å resolution. The present study shows that the benzimidazole ring of the inhibitor takes the stable stacking interaction with the protonated His57 in the catalytic domain of human chymase.

## 1. Introduction

Chymase is a mast-cell-specific serine protease that is stored within secretory granules and released together with heparin and histamine in response to allergen challenge or other stimuli. Recent studies have shown that chymases possess processing activity for biological peptides and cytokines implicated in a variety of diseases. For example, the chymases of primates and dogs have highly specific angiotensin II (Ang II) generating activity, and the results of animal studies suggest that chymase contributes to the pathogenesis of cardiovascular diseases *via* Ang II generation (Urata *et al.*, 1990 ▶; Takai *et al.*, 1997 ▶). Based on the results of these studies, inhibition of chymase is expected to provide therapeutic means for the treatment of these diseases.

An early approach toward the design of potent inhibitors for chymase has been to develop molecules containing activated ketones that achieve potency through the formation (or likely formation) of covalent adducts with the Ser195 or His57 residues of the catalytic triad (Aoyama *et al.*, 2001 ▶; Akahoshi *et al.*, 2001 ▶). Selectivity in this type of inhibitor becomes critical and such inhibitors with large molecular weight tend to show a lack of the chances of obtaining oral availability and minimal toxicity. This is prompting the search for non-covalent reversible inhibitors. There are relatively few reports describing inhibitors that specifically and rationally exploit non-covalent interactions with the common catalytic residues of chymase.

**Figure 1:**
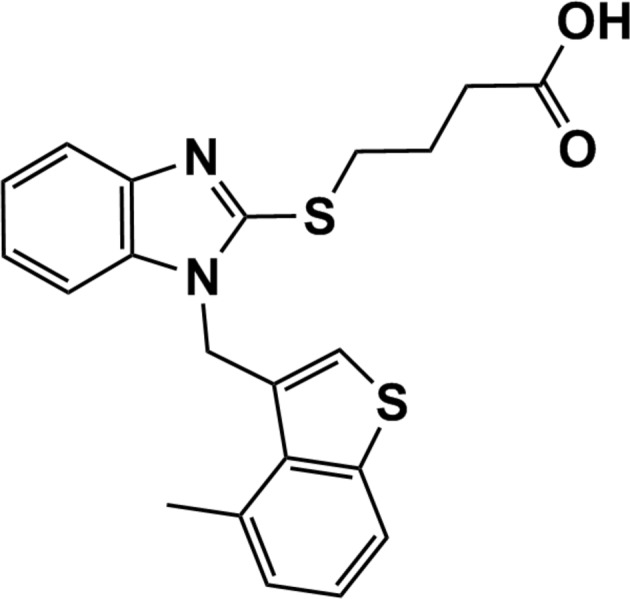
Benzimidazole derived human chymase inhibitor, TJK002.

Recently, we have developed some novel benzimidazole derived human chymase inhibitors and determined the crystal structures of the human chymase. The benzimidazole inhibitor TJK002 (Fig. 1 ▶; Yajima *et al.*, 2012 ▶) showed potent inhibitory activity (*K*_i_ value 2.24 n*M*) with respect to human chymase. The crystal structure of human chymase with TJK002 was determined at 2.8 Å resolution. X-ray crystallographic study showed that TJK002 forms a non-covalent interaction with the catalytic domain of human chymase. The 4-methylbenzothiophen-3-yl fragment of TJK002 occupies the S1 pocket. The carboxylic acid fragment of TJK002 forms hydrogen bonds with the imidazole N(∊) atom of His57 and/or the O(γ) atom of Ser195 which is a member of a catalytic triad. This imidazole ring of His57 forms π–π-stacking with the benzene ring of the benzimidazole scaffold as P2 moiety (Takenaka *et al.*, 1984 ▶). Fragment molecular orbital (FMO) calculation of the atomic coordinates by X-ray crystallography showed that this imidazole ring of His57 could be protonated with the carboxyl group of Asp121 or the oxyanion group of Ser195 and the stacking interaction between the benzimidazole group and His57 is stabilized. We propose a new drug design strategy using the stacking interaction of the protonated imidazole with the inhibitor causing unpredicted potent inhibitory activity even for other drug targets.

## 2. Materials and methods

### 2.1. Crystallization

The crystallization experiment of human chymase with TJK002 was performed using the hanging-drop vapour-diffusion method. The ligand was added to aliquots of the purified protein in a five-fold molar excess. Crystallization conditions were similar to those for the PMSF-treated human chymase crystal by mixing 1 µl of 6 mg ml^−1^ protein solution with an equal volume of the reservoir solution, which contained 100 m*M* sodium citrate (pH 5.5), 15% PEG1500 and 20% 2-propanol, and equilibrating against 1 ml reservoir solution (McGrath *et al.*, 1997 ▶). Single crystals grew to suitable dimensions in 2–4 d. Prior to flash-freezing in liquid nitrogen, the human chymase crystal was transferred to the reservoir solution with 30% glycerol and cooled at 79 K.

### 2.2. Data collection and structure determination

Diffraction data were collected on beamline NW12 at the Photon Factory (PF) and processed using the *HKL2000* software package (Otwinowski & Minor, 1997 ▶). Molecular replacement was performed using *MOLREP* (Vagin & Teplyakov, 1997 ▶) from CCP4 (Collaborative Computational Project, Number 4, 1994 ▶) with the coordinates of the PMSF-treated human chymase (PDB code 1klt; the solvent molecules and PMSF were removed) as the initial model. Refinement was carried out using the program *REFMAC* (Murshudov *et al.*, 1997 ▶). A sample containing a random 5% of the total reflections in the data set was excluded for *R*_free_ calculations. After rigid-body refinement, the electron density for the TJK002 ligand was clearly constructed using *COOT* (Emsley & Cowtan, 2004 ▶). In the final refinement at 2.8 Å, the crystallographic *R*_factor_ and *R*_free_ were 26.8 and 32.1%, respectively. Statistics of the data collection and final structure are summarized in Table 1 ▶. Figures were produced using *DS Visualizer* (Accelrys, http://accelrys.co.jp/).

### 2.3. FMO and molecular orbital energy calculation

The atomic coordinates of the complex used for FMO calculation used the complemented structure at the defected outer loop of the present chymase TJK002 crystal structure (PDB code: 4kp0). Construction of the defected loop and H atoms were generated using the *Discovery Studio* software, version 3.5.0 (Accelrys, San Diego, CA, USA). The CHARMM force field implemented in *Discovery Studio* version 3.5.0 was used for the minimization steps. Protein structures were optimized with the heavy atoms constrained of the crystal structure besides the inserted loop region. Using the FMO method, a molecule or molecular cluster is divided into *M* fragments (monomers). *Ab initio* calculations are then performed repeatedly on these monomer fragments in the presence of the electrostatic potential created by surrounding (*M* − 1) monomers (*V*_*J*_), until all the monomer densities become consistent. Next, the dimer equations are solved in the presence of the electrostatic potential from neighbouring (*M* − 2) monomers (*V* ^*IJ*^). Finally, the total energy of the system, *E*, is written as equation (1) using the total energies of the monomer *E*_*I*_ and the dimer *E*_*IJ*_,
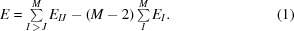
The inter-fragment interaction energy (IFIE) in the FMO calculations are defined by
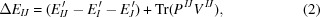
where *P* ^*IJ*^ is the differential density matrix, *V* ^*IJ*^ is the environmental electrostatic potential for the dimer, and 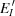 and 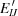 are the energies of the monomer and the dimer, respectively, in the absence of an environmental electrostatic potential. In this study, human chymase is divided into single amino acid residues to investigate intermolecular interactions between the enzyme and TJK002. The fragmented residues did not exactly correspond to each amino acid residue because partitioning of the chemical structure in the FMO scheme was performed between the Cα atom and the main-chain carbonyl group. Thus, the main-chain carbonyl group of the *i*th residue was assigned to the (*i*+1)th residue fragment. Using such fragmentation, each IFIE is regarded as a residue–residue interaction energy. Single-point energy calculations were performed by Hartree–Fock (HF) and second-order Møller–Plesset perturbation (MP2) methods (Fedorov & Kitaura, 2009 ▶), using the 6-31G basis set (FMO-HF/6-31G and FMO-MP2/6-31G). All FMO calculations were performed with the *ABINIT-MP* (version 5.0) program and the results visualized with *Biostation Viewer* (version 8.0) (CISS Free Software: http://www.ciss.iis.u-tokyo.ac.jp/english/dl/). Calculations were carried out on a DELL PowerEdge 1950III (Quad-Core Xeon X5470 3.33 GHz × 2) cluster.

We extracted only TJK002 and His57 coordinates from the present crystal structure and calculated molecular orbital energy using the quantum chemistry DFT/B3LYP method (*Jaguar*, version7.9: http://www.schrodinger.com/). The basis-set function used in the calculation was 6-31G**.

### 2.4. Determination of *K*_i_

*K*_i_ values for human chymase inhibition were determined using Suc-Ala-His-Pro-Phe-pNA (Bachem). Enzyme activities were evaluated by measuring *p*-nitroaniline release from the synthetic substrates. *K*_i_ values were determined by secondary plot methods using enzyme activities in some inhibitor and substrate concentrations.

## 3. Results and discussion

### 3.1. Binding conformation and hydrophobic interaction of TJK002

**Figure 2:**
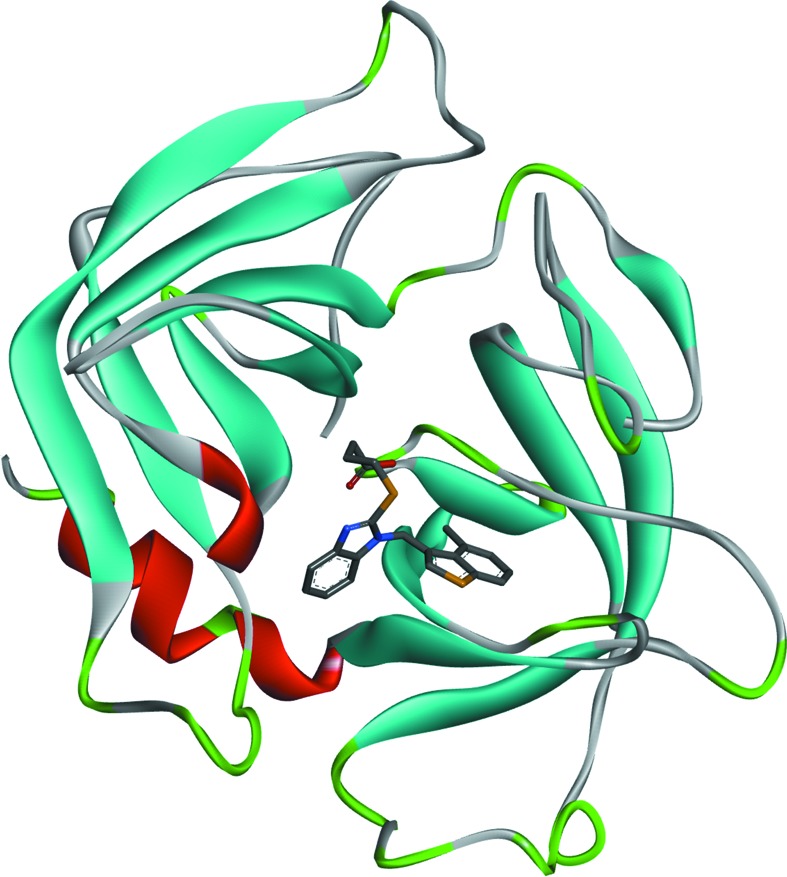
Overall structure of the human chymase TJK002 complex.

**Figure 3:**
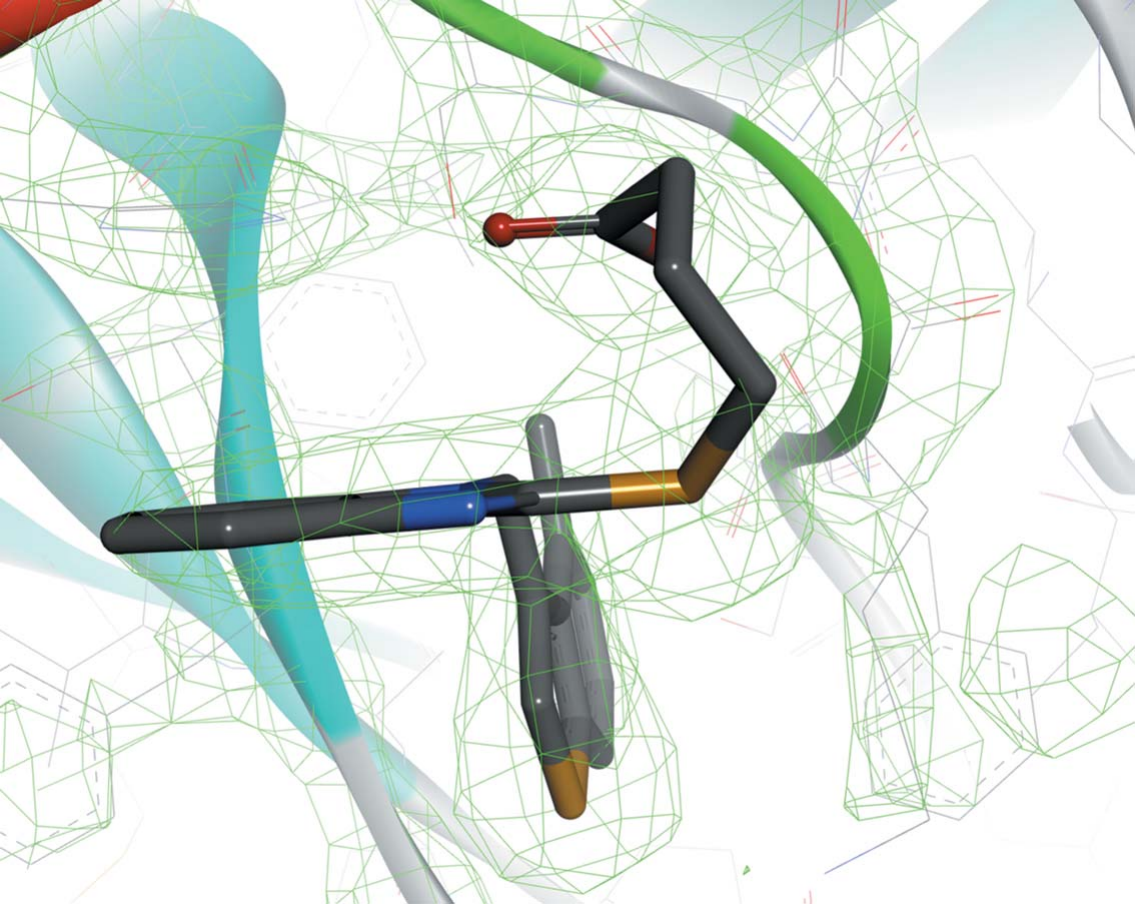
Crystal structure of TJK002 bound to the chymase active site.

The benzimidazole scaffold inhibitor TJK002 showed potent inhibitory activity (*K*_i_ value of 2.24 n*M*) with respect to human chymase. The crystal structure of the human chymase with TJK002 was determined at 2.8 Å resolution (Fig. 2 ▶). The X-ray co-crystal structure of TJK002 bound to the active site of human chymase is detailed in Fig. 3 ▶. X-ray crystallographic study showed that TJK002 forms a non-covalent interaction with the catalytic domain of human chymase. In particular, the 4-methylbenzothiophen-3-yl fragment of TJK002 occupies the S1 pocket as shown in Fig. 3 ▶. It is clear that the potent in­hibitory activity that TJK002 showed is derived from this effective hydrophobic interaction. The benzene ring of the benzimidazole scaffold shallowly occupies the S2 domain and could not form an effective hydrophobic interaction to the S2 site.

### 3.2. Oxyanion hole interaction of TJK002

**Figure 4:**
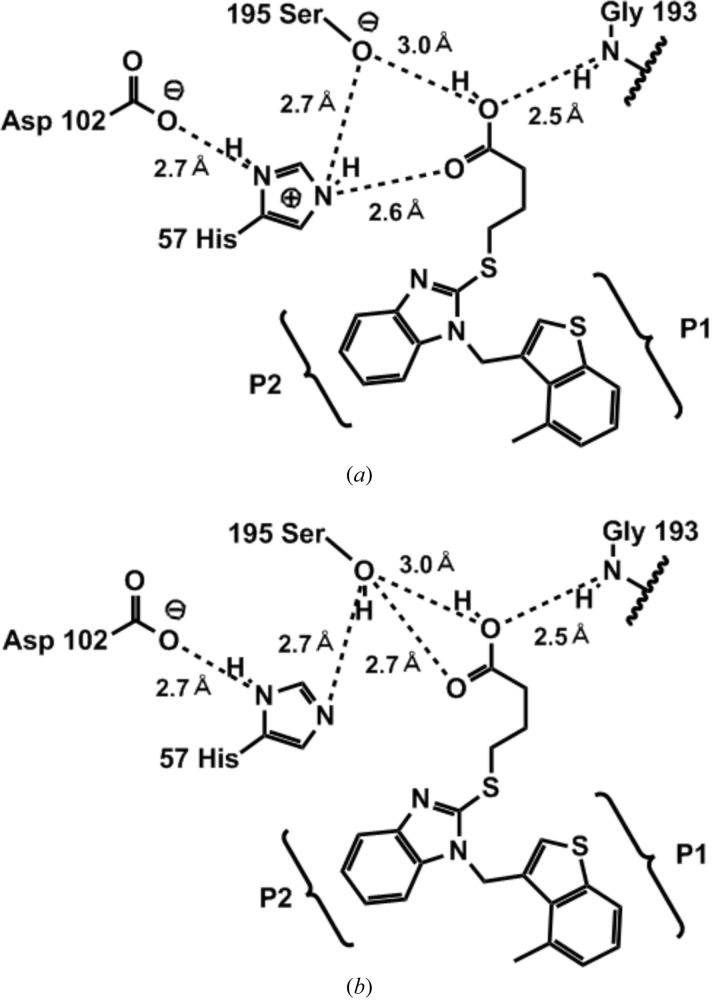
Depiction of the principal interactions: (*a*) protonated His57; (*b*) neutral His57.

The various close contacts that lead to important ligand–protein interactions are shown in Fig. 4 ▶. The alkyl chain of *S*-butyric acid is bent and the carboxylic acid motif of TJK002 forms hydrogen bonds to the residues which are members of a catalytic triad (His57, Ser195). There are known serine protease inhibitors that possess phosphonate or phosphinate groups, which can occupy the active site in the vicinity of the catalytic residues Ser195 and His57 (Greco *et al.*, 2007 ▶). However, phosphonate or phosphinate groups are known as undruglike motifs in the viewpoint of the pharmacokinetics profile. In our case the druglike carboxylic acid motif of TJK002 supplies two O atoms in the binding to the protein, which represent a new serine protease inhibitor motif. The position of this carboxylic acid motif is occupied by a water molecule in the case of another benzimidazole scaffold in­hibitor (Lo *et al.*, 2011 ▶).

**Figure 5:**
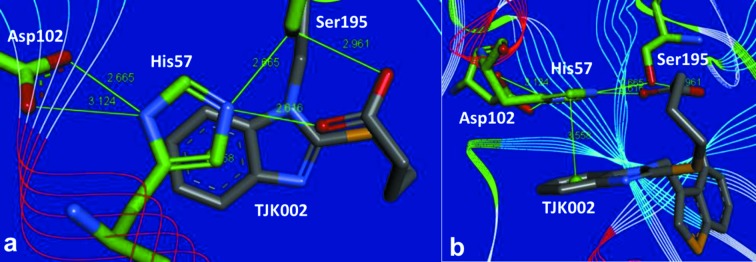
π–π-stacking interaction between ligand and protein. (*a*) Perpendicular view; (*b*) side view.

There are two possibilities from this crystal structure, protonated His57 state (Fig. 4*a* ▶) and neutral His57 state (Fig. 4*b* ▶). In the former state, His57 is protonated by Ser195 (Kawamura *et al.*, 2011 ▶). It is difficult to determine the proton position by X-ray analysis. The imidazole ring of His57 forms π–π-stacking to the benzene ring of the benzimidazole scaffold as shown in Fig. 5 ▶. So far the importance of the S2 site has not been noted, but this π–π interaction between the benzene ring of the benzimidazole scaffold inhibitor at the S2 site makes sense because the stacking interaction is also found in the complexes crystallized under different pH conditions (Lo *et al.*, 2011 ▶). The FMO calculation was performed in order to understand the features of this π–π-stacking interaction.

### 3.3. His57 stabilization with TJK002 by FMO and molecular orbital energy calculation

**Figure 6:**
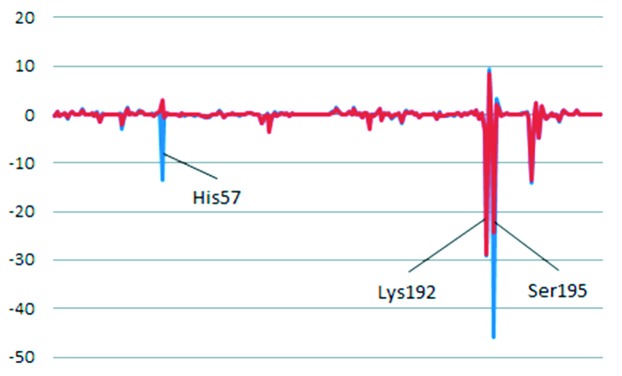
FMO calculation results. Energy in kcal mol^−1^. Blue: protonated His57. Red: neutral His57.

Fig. 6 ▶ shows the IFIE values between human chymase and TJK002 in the case of protonated His57 and neutral His57. The protonated His57 has a more stable IFIE value (−13.48 kcal mol^−1^) than the neutral His57 (+2.94 kcal mol^−1^) by binding of TJK002. FMO calculation of the atomic coordinates by X-ray crystallography showed that this imidazole ring of His57 could be protonated through hydrogen bonding with the carboxyl group of Asp102 or hydroxyl group of Ser195, and the stacking is stabilized in the crystallization performed at pH 5.5. The distance shows the strong hydrogen-bonding interaction between the N∊ atom of the protonated His57 and the carbonyl oxygen of the TJK002 (2.6 Å, shown in Fig. 4 ▶). This direct interaction could explain the potent inhibitory activity of TJK002. In order to understand the interaction energy, a FMO calculation was performed with His57 in its protonated and neutral states. The result clearly shows that even the neutral His57 shows a slightly repulsive IFIE value with TJK002, but TJK002 shows a dominant interaction with the protonated His57, similar to Lys192 of the S1 pocket and Ser195 as an oxyanion state. This result suggests that the protonated His57 is the probable state and the interaction with His57 plays an important role in the binding of TJK002 at the S2 site at physiological pH in the human body, the same as for S1 site. A similar stacking interaction between His57 and the benzimidazole ring is shown in human chymase complex which was crystallized at pH 8.0 (Lo *et al.*, 2011 ▶) in spite of the carboxyl group interactings with Lys192 not His57. His57 should be protonated at physiological pH.

**Figure 7:**
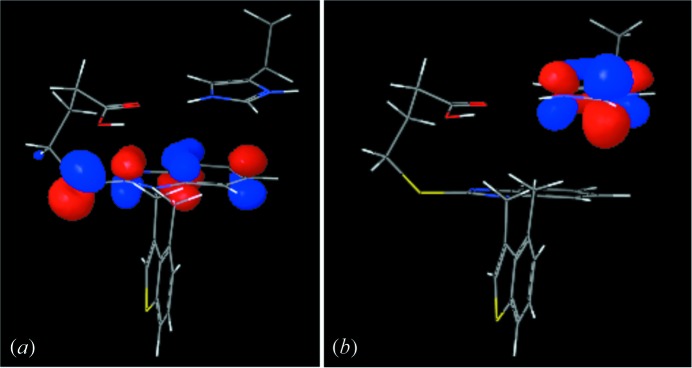
Result of the molecular orbital energy calculation. (*a*) NHOMO; (*b*) LUMO.

Fig. 7 ▶ shows NHOMO and LUMO orbitals and Table 2 ▶ shows the energy values of NHOMO, HOMO, LUMO and NLUMO. LUMO corresponds to the protonated imidazole ring and NHOMO corresponds to the benzimidazole ring. The energy gap between LUMO and NHOMO is 3.764 eV. This might be a possible value for the charge transfer through π–π-stacking interaction (Wu *et al.*, 2008 ▶). The π-orbitals of both rings have suitable symmetry and overlap efficiently.

## 4. Conclusion

We have found a novel, potent and non-covalent small-molecule human chymase inhibitor. The crystal structure of TJK002 bound to chymase shows (i) that potent inhibitory activities are derived from effective hydrophobic interaction to the S1 pocket of chymase, (ii) drug-like carboxylic acid acts as a new serine protease inhibitor motif and effectively interacts with the catalytic triad residues, (iii) the benzene ring of benzimidazole scaffold forms a π–π-stacking interaction with the protonated His57 that could compensate for the lack of hydrophobic interaction efficacy between the benzene ring of the benzimidazole scaffold and S2 site and also be used as the dominant guiding force for the potent inhibitory activity of this molecule. We propose a new drug design strategy using the stacking interaction of the protonated imidazole with the inhibitor causing the unpredicted potent inhibitory activity even against other drug targets.

## Supplementary Material

PDB reference: 4kp0

## Figures and Tables

**Table 1:** Data collection and refinement statistics for chymase/TJK002 Values in parentheses are for the highest-resolution shell.

Space group	*P*4_3_2_1_2
Unit-cell parameters (Å)	
*a*	56.9
*b*	56.9
*c*	177.5

Data collection	
Beamline	NW12 (PF)
Wavelength (Å)	1.0000
Resolution (Å)	50.0–2.80
Total number of reflections	211779
Unique reflections	27558
*R*_merge_	0.068 (0.422)
Completeness (%)	100.0 (100.0)
Multiplicity	15.2 (13.9)
*I*/σ(*I*)	40.7 (5.05)

Refinement statistics	
Resolution (Å)	41.0–2.80
*R*_factor_ (%)	24.2
*R*_free_ (%)	33.0
RMS deviation from ideal values	
Bond length (Å)	0.012
Bond angle (°)	1.70

*R*_merge_ = ΣΣ*i*|*I* *i* − 〈*I*〉|/Σ〈*I*〉, where 〈*I*〉 is the mean intensity of *N* reflections with intensities *I* *i* and common indices *h*, *k* and *l*. *R*_factor_ = Σ*_hkl_*||*F*_obs_| − *k*|*F*_calc_||/Σ_*hkl*_|*F*_obs_|, where *F*_obs_ and *F*_calc_ are the observed and calculated structure factors, *R*_free_ is calculated for a randomly chosen 5% of reflections and *R*_factor_ is calculated for the remaining 95% of reflections.

**Table 2:** Result of molecular orbital energy calculation (DFT/B3LYP method, 6-31G**)

	Energy (Hartree)	Energy gap
NHOMO	−0.293332	
HOMO	−0.285330	
LUMO	−0.155133	0.138 × 26.2116 = 3.764 eV
NLUMO	−0.126900	
